# Identification and functional validation of the *BcFPA*, a candidate regulator of flowering time in pak choi

**DOI:** 10.1186/s12870-026-09561-z

**Published:** 2026-07-28

**Authors:** Weihua Tang, Prosper Ndericimpaye, Qi Pan, Meiqing Jiang, Shutong Zhou, Xinran Zhao, Ying He

**Affiliations:** 1 Department of Agronomy and Horticulture, Jiangsu Vocational College of Agriculture and Forestry, Jurong, Jiangsu 212400 China; 2 Engineering and Technical Center for Modern Horticulture, Jiangsu Vocational College of Agriculture and Forestry, Jurong, Jiangsu 212400 China; 3 National Key Laboratory of Crop Genetics & Germplasm Innovation and Utilization, Key Laboratory of Biology and Genetic Improvement of Horticultural Crops (East China), Ministry of Agriculture and Rural Affairs of China, Engineering Research Center of Germplasm Enhancement and Utilization of Horticultural Crops, Ministry of Education of China, Nanjing Agricultural University, Nanjing, 210095 China; 4 Aksu Vocational and Technical College, Xinjiang Uygur Autonomous Region, Aksu, 843000 China

**Keywords:** Pak choi, Flowering time, QTL mapping, BSA-seq, Candidate gene

## Abstract

**Background:**

Flowering time is an essential complex quantitative agronomic trait in pak choi (*Brassica rapa* ssp. *chinensis*). It exerts decisive effects on crop yield, commercial quality and environmental adaptability throughout the whole cultivation cycle. Uncovering the key genes controlling flowering time is therefore critical for molecular genetic research and genetic improvement of pak choi.

**Results:**

In the present work, we performed bulked segregant sequencing (BSA-seq) using an F_2_ segregating population derived from a cross between late-flowering inbred line M1-006 and early-flowering line M2-001 to isolate and functionally characterize the flowering-related gene. Finally, a major-effect QTL governing flowering time was mapped to a 2.62 Mb genomic interval on chromosome A09, harboring a total of 440 annotated genes. Further gene function annotation pinpointed *Brap0046500.1* (designated *BcFPA*), an ortholog of the autonomous-pathway flowering regulator *AtFPA* from *Arabidopsis thaliana*, as the promising candidate gene within this target region, which was subsequently verified via polymorphic InDel molecular markers and genotype screening of F_2_ individuals. Combined sequence comparison and functional assays demonstrated that a non-synonymous substitution in the coding region of *BcFPA* leads to delayed floral transition in pak choi. Consistently, transgenic complementation assays in *Arabidopsis fpa-1* loss-of-function mutants verified that the wild-type *BcFPA* allele was sufficient to rescue the late-flowering phenotype and restore normal flowering. Moreover, overexpression of *BcFPA* in transgenic pak choi significantly accelerated flowering, further corroborating its positive regulatory role in floral initiation.

**Conclusions:**

This research systematically reveals the genetic mechanism responsible for natural flowering time variation in Brassica rapa. The identified *BcFPA* gene and its linked molecular markers can serve as important genetic resources for marker-assisted selective breeding, facilitating the breeding of new pak choi varieties with optimal flowering traits.

**Supplementary Information:**

The online version contains supplementary material available at https://doi.org/10.1186/s12870-026-09561-z.

## Background

Pak choi, also known as non-heading Chinese cabbage (*Brassica rapa ssp. chinensis*)is an important leafy vegetable cultivated widely in China and other East Asian countries for its nutritional and economic value [1]. Flowering time is a key agronomic trait affecting yield and commercial quality in pak choi [2]. However, premature bolting frequently occurs during pak choi production under fluctuating environmental conditions, resulting in reduced yield and commercial quality and limiting production scheduling. In contrast, excessively delayed flowering may reduce seed production efficiency [3, 4]. Flowering time also influences plant adaptation to environmental conditions [5]. Therefore, identification of genes associated with flowering-time regulation is important for understanding the genetic basis of this trait and for breeding pak choi cultivars with suitable bolting and flowering characteristics.

Flowering time is regulated by both genetic and environmental factors [6]. The genetic pathways regulating flowering time have been extensively characterized in the model plant *Arabidopsis thaliana*. Flowering-time regulation in *Arabidopsis* involves multiple pathways, including photoperiod, ambient temperature, vernalization, gibberellin, autonomous, and age-dependent pathways [7]. Flowering-time regulation has also been investigated in several crop species, including peanut (*Arachis hypogaea*) [8], rice (*Oryza sativa*) [9], radish (*Raphanus sativus*) [10], and maize (*Zea mays*). Multiple flowering-time regulators have been identified in these species. Many of these genes are homologous to flowering regulators identified in *Arabidopsis*, suggesting that flowering pathways are largely conserved among plant species. These flowering regulators function through distinct genetic pathways. For example, *GIGANTEA* (*GI*) [11], *FLOWERING LOCUS T* (*FT*), and *CYCLING DOF FACTOR 1* (*CDF1*) are mainly involved in the photoperiod pathway [12]. *FLOWERING LOCUS C* (*FLC*), *FRIGIDA* (*FRI*), and *VERNALIZATION INSENSITI 3* (*VIN3*) are associated with vernalization pathway [13]. In contrast, the autonomous pathway functions largely independently of environmental cues. *UMINIDEPENDENS* (*LD*), *FLOWER LOCUS PA* (*FPA*), *MULTICOPY SUPPRESSOR OF IRA 1* (*MSI1*), *PERPETUAL FLOWERING* (*PEP*), *FLOWER LOCUS CA* (*FCA*), and *FLOWER LOCUS K* (*FLK*) regulate flowering partly through repression of *FLC* expression within the autonomous pathway [14]. Among these genes, *FPA* is an important regulator of flowering time. For example, Loss of *FPA* function in *Arabidopsis* results in delayed flowering accompanied by elevated *FLC* expression [15]. *CpFPA1* was reported to promote early flowering in *Chimonanthus praecox* [16]. *AhFPA1* was also associated with earlier flowering in peanut(*Arachis hypogaea*) [8]. These studies suggest that *FPA* homologs are involved in flowering-time regulation in multiple plant species.

In Brassica species, numerous flowering-time quantitative trait loci (QTLs) have been identified across multiple chromosomes. In *B. napus*, more than 20 flowering-time QTLs were identified using a double haploid (DH) population [17], and *BnaFT.A02* and *BnaFLC.A02* were identified as candidates for vernalization-related variation by QTL-seq [18]; more recently, *BnaFLC.A10*, *BnaFLC.A02*, and *BnaFT.A02* were shown to jointly regulate flowering time [19]. In *B. rapa*, three flowering-time QTLs were detected, of which *FLQTL-1* was significant and contained *BrFLC2* [20]. In *B. oleracea*, eleven QTLs for flowering time were distributed across five chromosomes [21], and *BoFLC2* was recently identified as a major gene controlling flowering time in cabbage [22]. Although *FLC* paralogs and photoperiod genes such as *FT* and *CO* have been extensively studied in Brassica, the molecular identity of causal genes underlying flowering-time QTLs and their regulatory mechanisms remain largely uncharacterized in pak choi-particularly for autonomous pathway regulators such as *FPA*, which have received comparatively little attention in leafy Brassica vegetables. This gap provides the primary motivation for the present study. Bulked segregant analysis coupled with next-generation sequencing (BSA-seq) has proven effective for rapid identification of genomic regions associated with agronomic traits, and transgenic complementation in Arabidopsis has been widely used to validate candidate gene function in Brassicaceae [20]. Forward genetic approaches including linkage mapping and QTL analysis in segregating populations have also contributed substantially to dissecting flowering-time traits in Brassica crops.

Advances in next-generation sequencing (NGS) technologies have substantially improved the efficiency of genetic analysis in plant breeding research. BSA coupled with NGS is an advantageous technique for pinpointing target genes linked to favorable traits and marker-assisted selection (MAS) [23]. By comparing allele frequency differences between phenotypically contrasting bulks, SNPs and InDels associated with target loci can be rapidly detected [24]. This approach enables rapid localization of candidate genomic intervals without extensive individual genotyping and facilitates identification of candidate genomic regions associated with complex traits. Consequently, BSA-seq (QTL-seq) has been widely used for rapid QTL identification for identifying genomic regions associated with important agronomic traits in multiple crop species [25].

Pak choi is an important non-heading leafy vegetable of the Brassicaceae family, with a high nutritional value and wide cultivation. Flowering time has a significant impact on yield and market quality, which make it an important trait for agricultural production. However, the limited knowledge of flowering-time regulators has hindered genetic studies of flowering-time control in pak choi and restricted the use of molecular markers in breeding programs. Hence, the knowledge of these regulatory mechanisms is of immense agricultural and economic value. Considering the important role of the *FPA* gene in controlling plant developmental transitions and flowering time variation, we used bulked segregant analysis sequencing (BSA-seq) for the systematic identification and functional validation of the *FPA* gene in pak choi. These findings provide insight into the genetic basis of flowering-time regulation in pak choi and useful information for future breeding of cultivars with suitable flowering characteristics.

## Methods

### Plant materials, mapping populations, and phenotypic evaluation

The current study used two parental inbred lines of *Brassica rapa* ssp. *chinensis*, M2-001 and M1-006. To create the F_1_ plants, M2-001, the receiver parent with a very early-flowering phenotype, was crossed with M1-006, the donor parent with a very late-flowering phenotype. The offspring (F_1_) were then self-pollinated, yielding 350 F_2_ individuals that were used for BSA-seq [26]. In September 2024, the plants were cultivated at the Jurong experimental farm in Zhenjiang, China. Field management was carried out in accordance with conventional procedures, and all plants were seeded in the greenhouse. Plants were grown under natural photoperiod conditions (approximately 10–12 h light during the growth period, with day temperatures of 18–25 °C and night temperatures of 10–18 °C). The date of the first flower opening for 50% of the plants in every group was recorded to evaluate the flowering phenotype in the two parent lines, the F_1_ population and the F_2_ segregation mapping population [27]. The number of days between the date of sowing and flowering (DSF) was known as flowering time. A chi-square (χ^2^) test was carried out on the F_2_ population for genetic analysis [28]. For chi-square analysis of Mendelian segregation, F_2_ plants were categorized as early-flowering (DSF < = 120 days), intermediate (120 < DSF < 141 days), or late-flowering (DSF > = 141 days), using thresholds approximately 1 SD from the F_1_ mean (133 days).

### DNA extraction and pool construction

Fresh young leaves from the parents and F_2_ populations were harvested individually during the flowering stage, frozen right away in liquid nitrogen, and kept in a freezer at -80 °C. The Plant Genomic DNA Kit DP305 (Tiangeng Biotech; Beijing, China) was used to extract the total genomic DNA from the fresh leaves in accordance with the manufacturer’s manual [29]. A NanoDrop One spectrophotometer (Thermo Scientific, United States) and electrophoresis on 1% agarose gels were used to evaluate the purity and concentration of DNA. To create early-flowering and late-flowering DNA bulks for BSA-seq, the genotypes of thirty extreme early-flowering and thirty extreme late-flowering plants from the F_2_ generation were chosen. BSA-seq tests were conducted on the DNA of mixed pool samples that passed the quality assessment [30].

### Bulked segregant analysis (BSA) by resequencing

The early-flowering and late-flowering bulks were used to create resequencing libraries for bulked segregant analysis. For bulk construction, the 30 earliest-flowering F_2_ individuals (119–123 days) and 30 latest-flowering individuals (138–141 days) were selected to maximize phenotypic contrast between bulks. Following the qualification of the library check, the various libraries were combined, and Jisi Huiyuan Biotechnology Corporation (Nanjing, China) uses the Illumina NovaSeq X Plus platform for PE150 mode sequencing. We eliminated the adapter sequences and filtered out the low-quality bases. The Burrows-Wheeler Aligner program (BWA v0.7.17-r1188) was then used to align high-quality clean reads to the non-heading Chinese cabbage reference genome (*B. rapa* NHCC001) [31]. The alignment files were sorted, displayed, and indexed with default settings in the Samtools v1.18 program. Duplicate reads were found and marked employing GATK v4.4.0 [32]. Using Samtools’ view and index functions, only correctly paired reads with good mapping quality were kept for subsequent analysis. To obtain high-confidence biallelic SNPs and InDels, variant calling was performed for each chromosome using GATK HaplotypeCaller. The resulting variant sets were then filtered using vcftools v0.1.16 (function: filter). Further filtering made sure that (1) distinct loci had biallelic variations, (2) homozygous loci had variation between parental lines or bulks, and (3) distinct loci on the main chromosome had sequencing depth ≥ 4× [8]. We found high-quality polymorphic SNPs/InDels loci after filtering. The SNP index and InDel index, which show the proportion of reads harboring the alternative allele at each SNP and InDel locus, were computed for both late- and early-flowering bulks. Subtracting the SNP/InDel index of the late-flowering bulk from the SNP/InDel index of the early-flowering bulk yielded the ΔSNP index and ΔInDel index. We identified a possible candidate genomic interval with flowering regulatory genes based on the ΔInDel index. With default settings, QTL-seq v2.2.3 (qtlplot) was used to visualize the ΔSNP-index and ΔInDel-index data. The genomic region that had ΔSNP-index or ΔInDel-index values within the 95% confidence interval was the only one that was selected [33].

### Candidate gene prediction and annotation

The non-heading Chinese cabbage database (*B. rapa* NHCC001) provided information on the putative coding genes’ annotation inside the candidate interval. Multiple databases, including the Kyoto Encyclopedia of Genes and Genomes (KEGG), Gene Ontology (GO), Swiss-Prot, the protein family (Pfam) database, and the nucleotide collection (NR), were also used. All annotated genes were examined based on their *Arabidopsis* homologs using the *Arabidopsis* database’s BLAST tool (TAIR, http://www.arabidopsis.org//Blast/index.jsp). Thus, *B. rapa* genes with a high level of similarity to *Arabidopsis* were selected for subsequent investigations [32, 34].

### Validation of InDel markers in the candidate region

InDel markers for PCR amplification were extracted from BSA-seq data to assess linkage between the candidate region and flowering time. These markers were used in a polymorphism analysis between early- and late-flowering genotypes. Then, a small population consisting of two parents, eight extremely late-flowering individuals, and eight extremely early-flowering individuals was analyzed using markers showing clear polymorphic banding patterns. 10 µL 2 × Rapid Taq Master Mix (Vazyme, Nanjing, China), 0.5 µL 10 µM forward and reverse primers each, 1 µL genomic DNA (100–300 ng), and 8 µL distilled water were used in a 20 µL volume for the PCR. Starting at 95 °C for 3 min, the thermocycling was followed by 35 cycles of 95 °C for 15 s, 55 °C for 15 s, and 72 °C for 5 s, with a final extension at 72 °C for 5 min. The primers used are listed in Table S6.

### Transgenic validation in *Arabidopsis* and pak choi

We used Agrobacterium-mediated transformation to examine the function of the candidate gene (*BcFPA*). A One Step Cloning Kit (Vazyme, Nanjing, China) was utilized to create the recombinant plasmids *35 S: BcFPA*^*wt*^ and *35 S: BcFPA*^*mut*^. This was accomplished by cloning the full-length coding sequences of *BcFPA*^*wt*^ and *BcFPA*^*mut*^ into the pCAMBIA2300 vector. The validated recombinant plasmid was introduced into GV3101 (*Agrobacterium tumefaciens*), which was then transformed into *Arabidopsis thaliana* using the floral dip process. This experiment followed the protocol that had previously been published for *Arabidopsis* [35]. The flowering phenotypes of the transgenic lines were compared with those of the *Landsberg erecta* (Ler) wild type and *fpa-1* mutant controls to assess functional complementation in *Arabidopsis*. Furthermore, *BcFPA*^*wt*^ overexpression was conducted in pak choi to investigate its regulatory function in floral development. For pak choi transformation, the cotyledon node explant method was used: cotyledon explants were co-cultivated with GV3101 harboring the *35 S::BcFPA*^*wt*^ construct; kanamycin selection (50 mg/L) was applied during regeneration on MS medium supplemented with 1.0 mg/L 6-BA and 0.1 mg/L NAA. T_0_ plants were confirmed by PCR of the *NptII* marker and *BcFPA* transgene; T_1_ plants were verified by qRT-PCR for *BcFPA* overexpression; all phenotypic analyses were performed using T_2_ homozygous lines to minimize insertion position effects. The primers used are listed in Table S6.

### RNA isolation and quantitative real-time PCR

In accordance with the manufacturer’s manual, total RNA was extracted from leaves using the Total RNA Extraction Reagent (Vazyme Biotech Co., Ltd., Nanjing, China). For transgenic pak choi, leaf tissue samples were collected at the 8–10 rosette leaf stage, prior to visible bolting or flower bud emergence, to ensure that expression analysis reflected pre-flowering regulatory states. RNA integrity was verified by 1.0% agarose gel electrophoresis, and RNA concentration and purity were assessed using a NanoDropTM One spectrophotometer set to 260 nm. Vazyme Biotech Co., Ltd.‘s HiScript^®^ II 1st Strand cDNA Synthesis Kit was used to perform first-strand cDNA synthesis. Using a Bio-Rad CFX96 Touch Real-Time PCR Detection System and the 2× ChamQ Blue Universal SYBR qPCR Master Mix (Vazyme Biotech Co., Ltd., Nanjing, China), quantitative real-time PCR (qRT-PCR) was carried out. Each 20 µL reaction used 0.4 µL of each primer, 2.0 µL of cDNA template (diluted at 1:10), 10 µL of 2×SYBR Master Mix, and ddH_2_O to fill the remaining volume. The following were the thermal cycling conditions: After four minutes of initial denaturation at 95 °C, 40 cycles of 10 s at 95 °C and 30 s at 60 °C were performed. As an internal reference, the *Brassica rapa* actin gene (*BraC03g019900*) was used to calculate relative gene expression levels using the 2^−ΔΔCt^ method. The Primer Premier 5 program was used to create primers for qRT-PCR. Each qRT-PCR test was run in triplicate using three biological replicates. Three technical replicates of each sample were used for measurement. Student’s t-test was used for all statistical analyses in SPSS 23.0 software to compare the differences between the transgenic and wild-type (WT) genotypes [36]. The primers used are listed in Table S6.

## Results

### Phenotypic evaluation and inheritance analysis of flowering in pak choi

**Fig. 1:**
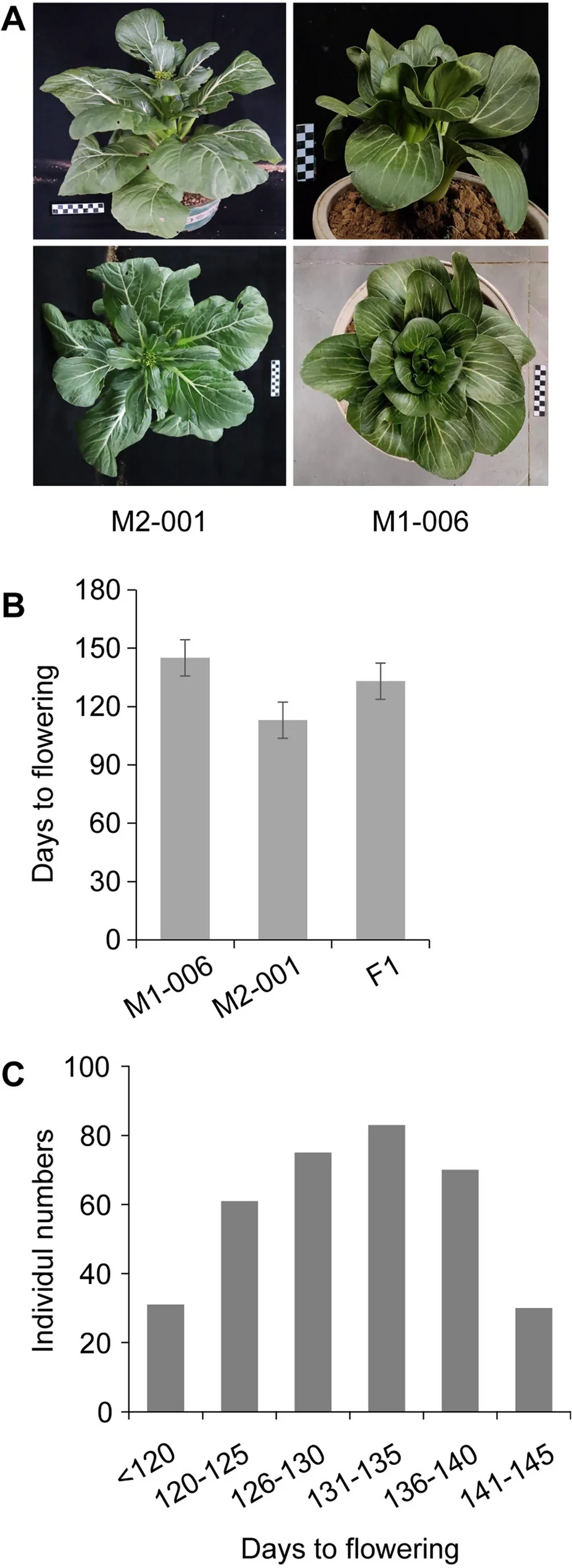
Phenotypic characterization and the distribution of flowering time in pak choi. **A** The two parental inbred lines employed in this investigation, with M2-001 representing the early flowering trait and M1-006 denoting late flowering. **B** The flowering times of parental inbred lines and F_1_ line. **C** The distribution of flowering times in the F_2_ population (*n* = 350)

To identify genomic regions associated with flowering time in pak choi, an F_2_ population was generated by crossing the early-flowering line M2-001 with the late-flowering line M1-006 (Fig. 1A). The late-flowering genotype M1-006 flowered on the 145th day from sowing to flowering (DSF), whereas M2-001 flowered on the 113th DSF (Fig. 1B). Both parental lines were cultivated at the Jurong experimental farm (Zhenjiang, China). The two genotypes’ average flowering dates differed significantly by 32 days. The F_1_’s mean bolting time was 133 DSF (Fig. 1B), which was somewhat higher than the mid-parental value of 129. Analysis of flowering time in 350 F_2_ individuals a continuous distribution, with extreme early-bolting and late-bolting exhibiting ranges of 120 to 141 DSF and an average of 132 DSF, respectively (Fig. 1C). Few plants exhibited the parental extreme phenotypes, and most intermediate plants made up the F_2_. The genetic assessment of F_2_ offspring revealed that the ratio of flowering did not match the expected Mendelian 1:2:1 pattern (Chi-Square test, *p* < 0.0001) (Table S1). These results indicate that flowering time is a quantitative trait controlled by multiple loci rather than a single Mendelian gene.

### Primary mapping of the flowering‑related QTL by BSA‑seq

**Fig. 2:**
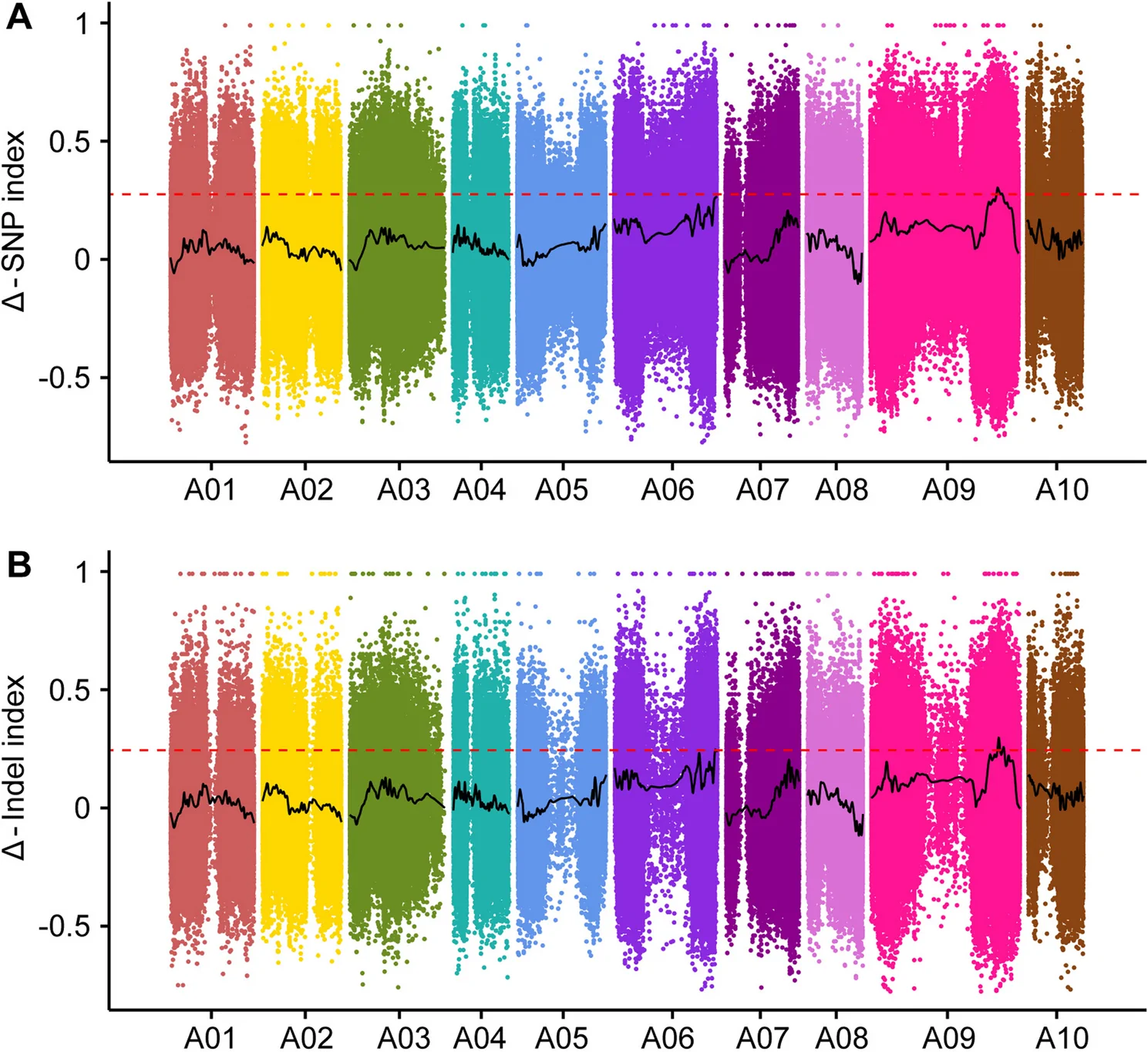
Results of the BSA-seq analysis. Based on BSA-seq analysis, the distribution of SNP-index and InDel-index was screened on the chromosome. Each colored block on the x-axis represents one of the ten *B. rapa* chromosomes, designated A01-A10. The delta SNP index (**A**) and delta InDel index (**B**), which illustrate the variation in SNP or InDel allele frequencies between the two bulks, are displayed on the y-axis. The candidate region was found on chromosome A09 between 55.33 and 57.95 Mb. Each dot represents an individual SNP/InDel position across the genome. ΔSNP-index values were smoothed using a 1-Mb sliding window with 10-kb increments

To identify genomic regions associated with flowering time, two phenotypically contrasting bulks were constructed from the F_2_ population Genomic DNA samples were grouped into an early-flowering bulk (M2-pool) and a late-flowering bulk (M1-pool). Equal amounts of DNA from 30 early-flowering and 30 late-flowering F_2_ individuals were pooled separately for BSA-seq analysis. BSA-seq generated 122,634,548 clean reads for the M1-pool and 108,799,806 clean reads for the M2-pool (Table S2). Approximately 97.09% and 97.06% of reads from the M1-pool and M2-pool, respectively, were successfully aligned to the *B. rapa* NHCC001 reference genome [31]. The Q30 values were 94.59% for the M1-pool and 94.45% for the M2-pool. Genome coverage at 1× depth reached 96.18% in the M1-pool and 96.25% in the M2-pool. At sequencing depths of 5× and 10×, genome coverage reached 93.47% and 90.72% in the M1-pool and 93.02% and 88.71% in the M2-pool, respectively (Table S2). After variant calling and filtering, 16,342 high-confidence SNPs and 4,073 InDels were identified on chromosome A09 (Table S3). Then, ΔSNP-index, and ΔInDel-index values were calculated by comparing the two bulks. A prominent ΔSNP-index peak was detected on chromosome A09 within a 2.62-Mb interval spanning 55.33–57.95 Mb. A prominent ΔInDel-index peak was detected on chromosome A09 within a 2.98-Mb interval spanning 55.22–58.20 Mb. The interval (A09: 55.33–57.95 Mb) was therefore considered a candidate genomic region associated with flowering-time variation (Fig. 2).

### Candidate gene prediction and functional annotation

Based on gene annotation of the *B. rapa* NHCC001 reference genome, a total of 440 annotated genes were distributed within the 2.62 Mb candidate interval on chromosome A09 (Table S4). To narrow down causal flowering-related genes from this large genomic segment, functional homology screening was performed by aligning all 440 interval genes against the Arabidopsis genome database. Only one gene, *Brap0046500.1*, was matched to the known flowering suppressor gene AT2G43410 (*AtFPA*). The remaining 439 genes either lacked Arabidopsis homologs or their Arabidopsis orthologs had no documented role in flowering-time regulation. Loss-of-function mutation of *AtFPA* leads to obvious late-flowering phenotype in Arabidopsis. In the model plant, *AtFPA* acts as a post-transcriptional regulator in the autonomous flowering pathway to accelerate floral transition mainly by suppressing *FLC* transcription through alternative polyadenylation and RNA 3′-end processing; this protein belongs to the RRM-SPOC family and is characterized by conserved RNA recognition motifs (RRMs) and a C-terminal SPOC domain [15]. Genome positioning revealed that *Brap0046500.1* resides at 56.333507–56.337767 Mb on chromosome A09 with a physical span of ~ 4.26 kb (Table S4). Taken together with its conserved functional features, *Brap0046500.1* was prioritized as the key candidate gene responsible for natural flowering-time variation in pak choi and subjected to follow-up functional characterization.

### Validation of InDel markers in the 4.26 kb interval and sequence variation analysis

**Fig. 3:**
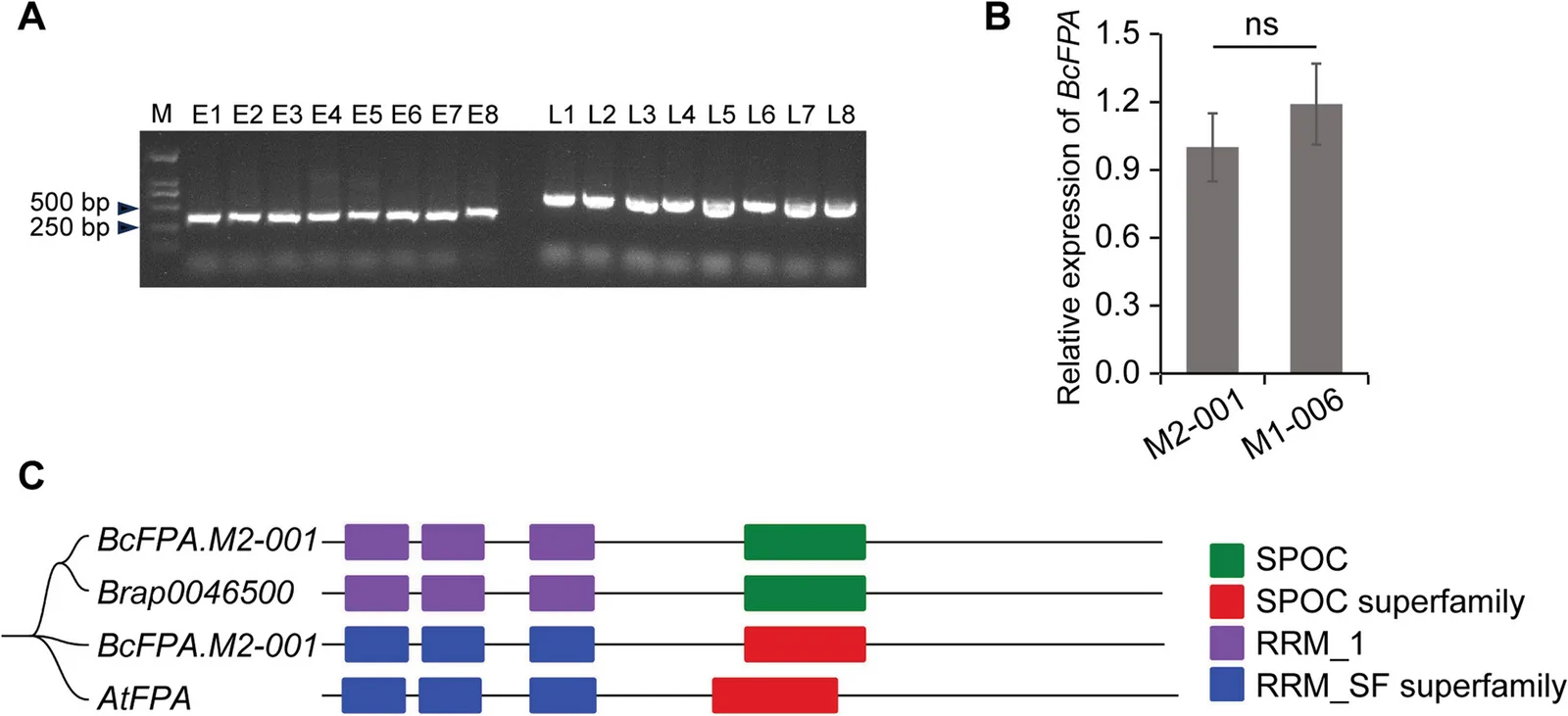
Co-segregation of an InDel marker with flowering time in extreme F_2_ population and domain analysis. **A** PCR results for an InDel marker that co-segregated with the extreme F2 population. M stands for marker, E for early flowering, and L for late flowering. The early-flowering allele produces a band of approximately 381 bp, while the late-flowering allele produces a band of approximately 666 bp. **B** Relative expression level of the BcFPA gene in two parental lines. ns means no significant difference based on Student’s t-test. Error bars represent mean ± standard deviation (SD). **C** Evolutionary relationship among FPA homologs in *Arabidopsis thaliana* and Pak choi. *BcFPA.M1-006* is from M1-006, *BcFPA.M2-001* is from M2-001, AtFPA is from *Arabidopsis thaliana* (AT2G43410), and *Brap0046500* is from NHCC001. The three RRM and SPOC domains are significantly conserved

To further verify whether this *Brap0046500.1* gene is associated with flowering phenotype, InDel markers within the 4.26-kb candidate interval containing *Brap0046500.1* (Table S5, Fig. S1-S2) were used to evaluate the association between this region and flowering-time phenotypes. A subset of 16 extreme F_2_ individuals, including 8 early-flowering and 8 late-flowering, were selected for InDel marker validation. According to the PCR amplification results, one InDel marker (Fig. S1-S2) had a robust and reproducible polymorphism between both parents and the secondary F_2_ progenies (Table S5). Using phenotypic data and the considerable degree of polymorphism, this InDel marker showed clear co-segregation with flowering-time phenotypes in the extreme F_2_ individuals (Fig. 3A, Table S6), confirming that the target genomic interval harbors the causal locus governing flowering-time variation.

As part of sequence comparison between the two parental lines, we characterized sequence polymorphisms within *Brap0046500.1* and identified two insertion mutations located in the final exon (Fig. S3), which was uniquely derived from the early-flowering parent M2-001. These mutations map specifically to the SPOC (Spen paralogue and orthologue C-terminal) domain-encoding region—a conserved domain critical for protein–protein interactions and transcriptional repression in SPEN family proteins. To clarify whether this variant affects gene transcription, we quantified its transcript abundance in parental lines at the flowering stage via qRT-PCR, and no significant expression difference was detected between genotypes (Fig. 3B). These findings imply that the insertion alters protein functionality instead of transcriptional abundance.

Phylogenetic analysis of FPA homologs from pak choi and *Arabidopsis thaliana* verified the orthologous relationship between pak choi *BcFPA* and *AtFPA*. The two allelic variants of *Brap0046500.1* from M2-001 and M1-006 were designated *BcFPA.M2-001* and *BcFPA.M1-006*, respectively, and phylogenetic clustering revealed close evolutionary relatedness of these two alleles (Fig. 3C). Conserved domain analysis demonstrated that both predicted proteins belong to the Split ends (Spen) family, featuring N-terminal RNA recognition motifs (RRMs) and a conserved C-terminal SPOC domain, consistent with the canonical domain architecture of AtFPA; specifically, three conserved RRM domains were identified in both allelic proteins (Fig. 3C). Collectively, these lines of evidence support *Brap0046500.1* as a candidate gene controlling flowering time in pak choi, which was renamed *BcFPA* hereafter, and subsequent functional validations were implemented to confirm its biological role.

### Functional verification of the candidate gene *BcFPA* in transgenic *Arabidopsis* and pak choi

**Fig. 4:**
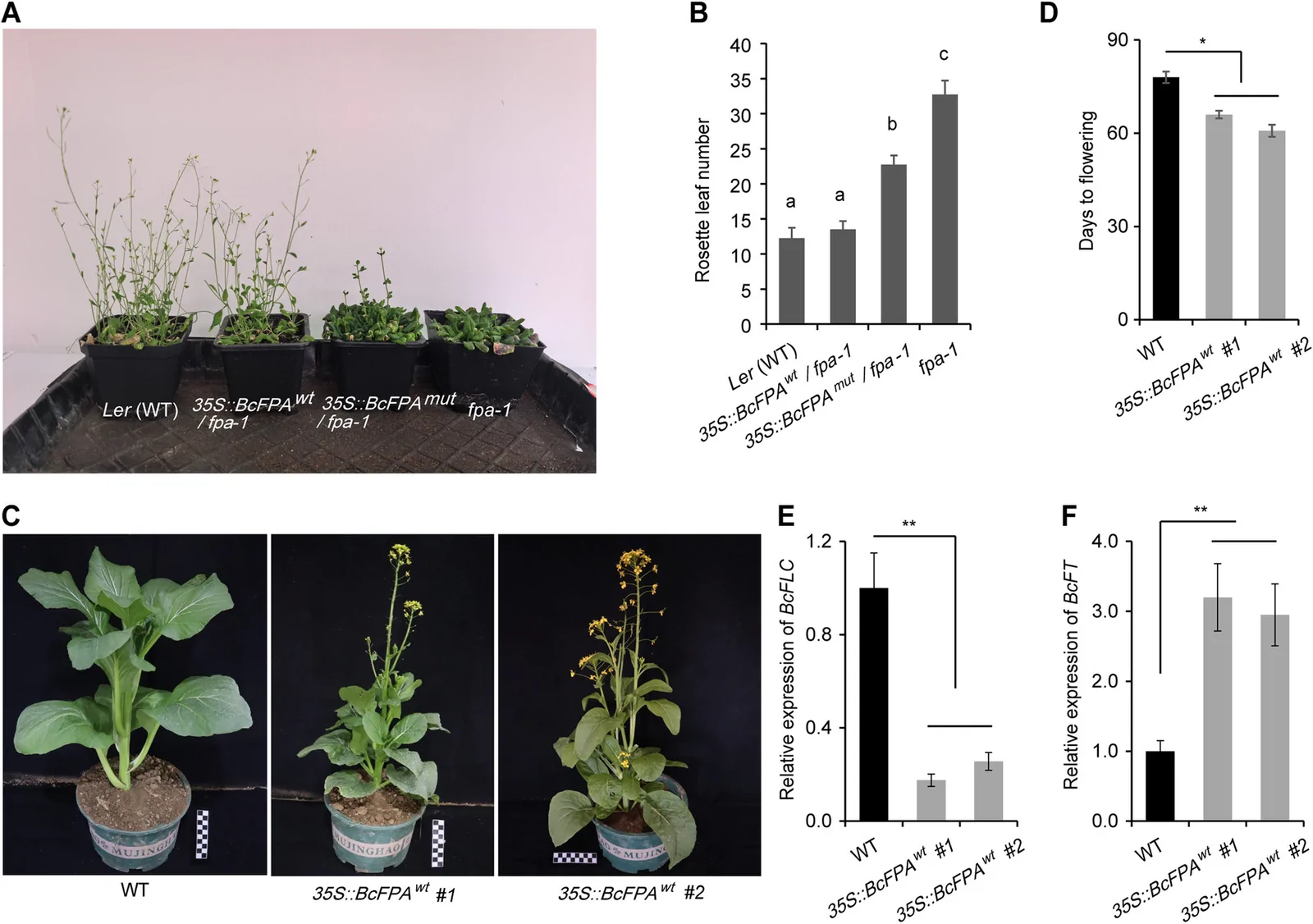
Functional validation of *BcFPA* in transgenic *Arabidopsis* and pak choi. **A** Evaluation of flowering phenotypes in Arabidopsis thaliana, encompassing Landsberg erecta (Ler) as the wild-type (WT), the fpa-1 mutant, and two transformed fpa-1 plants (*35S: BcFPA*^*wt*^ and *35S: BcFPA*^*mut*^). *BcFPA*^*wt*^ refers to the functional allele isolated from the early-flowering parent M2-001. *BcFPA*^*mut*^ refers to the allele from the late-flowering parent M1-006. The floral dip technique was employed to transform the plants. **B** Quantification of flowering time by total leaf number at bolting under long day. Means ± SD were calculated from N = 10 individuals. **C** Comparison of the wild-type pak choi to the transgenic plant that overexpresses BcFPA (*35S: BcFPA*^*wt*^). **D** Quantification of flowering time by days to flowering under long day. Means ± SD were calculated from *N* = 6 individuals. **E**-**F** The relative expression of flowering-related genes (*BcFLC* and *BcFT*) in both wild-type and *BcFPA*-overexpressing plants. Plants that overexpress *BcFPA* have significantly reduced BcFLC expression and elevated *BcFT* expression. The findings are shown as mean ± SD. All data was analyzed using the student’s t-test. The significant differences are shown by one asterisks (*P* < 0.01) and double asterisks (*P* < 0.001)

To characterize the biological function of *BcFPA*, we introduced its wild-type (*35 S: BcFPA*^*wt*^) and mutant (*35 S: BcFPA*^*mut*^) alleles into the *Arabidopsis fpa-1* loss-of-function mutant via Agrobacterium-mediated transformation driven by the CaMV 35 S constitutive promoter, with wild-type plants serving as controls. Phenotypic observations revealed that ectopic expression of *BcFPA*^*wt*^ largely rescued the late-flowering defect of *fpa-1* and recovered wild-type flowering behavior, whereas transgenic lines overexpressing *BcFPA*^*mut*^ retained a delayed-bolting phenotype identical to the untransformed mutant (Fig. 4A and B). These results confirm that the identified sequence mutation impairs the physiological function of *BcFPA* and validate its essential role in flowering-time control.

To further evaluate the potential role of *BcFPA* in flowering-time regulation, the *BcFPA* overexpression construct was introduced into pak choi using Agrobacterium-mediated transformation. Compared with wild-type plants, transgenic lines overexpressing *BcFPA* exhibited earlier flowering phenotypes (Fig. 4C and D). Transgenic plants flowered at approximately 66 days from sowing, whereas wild-type plants flowered at approximately 78 days from sowing. Expression analysis of flowering-related genes showed that *BcFLC* expression was reduced in transgenic plants (Fig. 4E). In contrast, expression of *BcFT* was significantly increased in BcFPA-overexpressing plants compared with wild-type plants (Fig. 4F). These results suggested that *BcFPA* is associated with flowering-time regulation in pak choi. The observed expression patterns of *BcFLC* and *BcFT* suggest that *BcFPA* may participate in flowering-time regulation through pathways like those reported in *Arabidopsis*.

## Discussion

Flowering time is an important agronomic trait that determines the transition from vegetative growth to reproductive development in plants. In leafy vegetables such as pak choi (*Brassica rapa* ssp. *chinensis*) flowering time influences biomass accumulation, production period, and seed yield. Numerous studies reported that genes related to flowering time control crop production in rapeseed [37], rice [38], and tomato [39].Although *FPA* has been reported to participate in flowering-time regulation in *Arabidopsis* and several other species, its role in *B. rapa* remains unclear. In this study, we combined BSA-seq analysis with transgenic experiments to investigate the potential involvement of *BcFPA* in flowering-time variation in pak choi. Using an F_2_ segregation population, we identified a flowering-time QTL within a 2.62-Mb interval on chromosome A09 and identified *Brap0046500.1* (*BcFPA*) as a candidate gene associated with flowering-time variation in pak choi. These findings provide insight into the genetic basis of flowering-time regulation in pak choi and provide genetic resources for future breeding of flowering-time traits in pak choi.

In this study, an F_2_ segregating population was used to investigate the genetic basis of flowering-time variation in pak choi. The two parental lines displayed clear differences in flowering time (Fig. 1), indicating that the flowering phenotype in pak choi is strongly controlled by genetics and that the two parents carry different alleles at the flowering time QTL [40]. The phenotypic divergence between the parental lines facilitated BSA-seq analysis and enabled the identification of candidate genomic regions associated with flowering time. Phenotypic analysis of the F_2_ population showed continuous variation in flowering time (Fig. 1C), indicating that flowering time is likely controlled by multiple loci. In *B. rapa*, *B. oleracea*, *and B. napus*, similar quantitative or genetic inheritance patterns have been reported, where flowering time is controlled by multiple loci with varying genetic effects [41–43]). Despite the quantitative nature of the trait, BSA-seq analysis identified a candidate interval on chromosome A09 with a prominent ΔSNP-index signal (Fig. 2), suggesting that this region may contribute substantially to flowering-time variation in flowering time between the early- and late-flowering lines. This candidate interval provides a basis for further investigation of flowering-time regulation in pak choi. Similar genetic architectures involving quantitative inheritance and major-effect QTLs, were reported in *Brassica rapa* [34], a hybrid of broccoli and cabbage [44], as well as other species such as *Cucumis sativus* [45], *Eucalyptus grandis* [46], *Panicum hallii* [47], *Juglans regia* [48], and *Arachis hypogaea* [8]. BSA-seq has been widely used to identify genomic regions associated with agronomic traits such as disease resistance [49, 50], plant height [51], branching [31], and flowering-related traits in multiple crops. QTL-seq was also used to identify multiple genes associated with important traits in pak choi, including *Brnye1* for the stay-green (non-yellowing) phenotype [52]; the *BrCHI3* and *BrLBD39* genes for the purple coloration [53]; and *BrGH3.12* and *BrABF1* for leaf shape [54]. In this study, extreme flowering-time bulks were constructed for genome-wide variant analysis using NGS data (early-flowering and late-flowering) and SNP-index analysis was subsequently used to identify candidate genomic intervals associated with flowering time. By sequencing F_2_ progeny with extreme phenotypic differences derived from two parental lines, this method enabled rapid identification of candidate regions and provided sequence variants for future fine-mapping studies [55].

Following identification of the candidate interval, functional annotation identified a single gene, *Brap0046500.1*, homologous to *FPA* (AT2G43410) in *A. thaliana—*a known regulator of flowering time in the autonomous pathway. Based on positional and functional evidence, *Brap0046500.1* was designated *BcFPA*. The identification of *BcFPA* is notable because, while *FLC* paralogs and photoperiod genes such as *FT* and *CO* have been extensively studied in Brassica, autonomous pathway regulators such as *FPA* have received comparatively less attention in leafy Brassica vegetables. Within the autonomous pathway, *AtFPA* promotes flowering by modulating *FLC* expression through RNA 3’-end processing and alternative polyadenylation of *FLC* antisense transcripts (*COOLAIR*), preventing accumulation of the *FLC* flowering repressor independently of day length or vernalization. *FPA* proteins belong to the SPEN family, characterized by N-terminal RNA recognition motifs (RRMs) and a C-terminal SPOC domain mediating protein–protein interactions. Downstream, reduced *FLC* expression permits activation of *FT* and *SOC1*, accelerating floral transition [15, 56]. Our results support *BcFPA* as a candidate gene contributing to this conserved pathway in pak choi. Similar BSA-seq and marker-based approaches have been used to identify candidate loci for agronomic traits in multiple crops; for example, Habila et al., [57] identified salt-tolerance QTLs in rice, Zhang et al., [58] identified *AhRt2* for red testa in peanut, and Zhang et al., [59] mapped *Brwf* for white flower in Chinese cabbage using similar strategies.

InDel marker analysis further confirmed the association of the *Brap0046500.1* locus with flowering-time phenotypes: one marker within the 4.26-kb candidate interval showed stable co-segregation with the early- and late-flowering phenotypes in selected F_2_ individuals (Fig. 3A), supporting the relevance of this genomic interval. Although only one informative marker was obtained, the combination of BSA-seq localization, marker co-segregation, and functional experiments provides converging evidence for *BcFPA* as a candidate gene. Sequence analysis identified non-synonymous mutations in the SPOC domain-encoding region of the final exon of BcFPA. Because SPOC domain integrity is critical for protein—protein interactions required for *FLC* repression, these mutations likely impair BcFPA function at the protein level—consistent with the absence of significant *BcFPA* transcript-level differences between parental lines (Fig. 3B). The non-synonymous nature of the mutations, rather than transcriptional changes, as the likely driver of phenotypic divergence is consistent with the delayed-flowering phenotype observed in the Arabidopsis *fpa-1* mutant and highlights the importance of combining sequence analysis, structural prediction, and functional experiments when investigating candidate genes underlying complex quantitative traits.

Transgenic complementation experiments in *Arabidopsis* suggested that *BcFPA* and its homolog *AtFPA* may have comparable functions in promoting early flowering. The transgenic plants carrying the wild-type allele (*FPA*^*wt*^) substantially restored flowering time in the *Arabidopsis fpa-1* mutant. whereas the mutant allele (*FPA*^*mut*^) failed to rescue the late-flowering phenotype (Fig. 4A). This observation supports the effect of a non-synonymous mutation on the phenotype [60, 61]. Numerous studies showed that flowering-related regulators, such as the *FLC*,* FT*, and *SOC1* genes, are functionally conserved in *Arabidopsis*, Brassicaceae, and many other plant species. Similarly, our research showed that these genes may be evolutionarily conserved and participate in the control of flowering time in pak choi. In transgenic pak choi, expression levels of *BcFPA* and *BcFT* were significantly increased in transgenic pak choi compared to the wild type, whereas *BcFLC* was reduced (Fig. 4E-F). These expression patterns are consistent with the proposed role of *BcFPA* in flowering regulation. These results are consistent with phenotypic data, where the transgenic plants (*BcFPA*^*wt*^) flowered significantly earlier than the wild types (Fig. 4C). Similar results were observed in *Arabidopsis thaliana* and peanut [8, 15, 62].

In this study, *BcFPA* was identified as a candidate gene associated with flowering-time variation in pak choi. This gene may provide a useful target for marker-assisted selection (MAS) or future genetic improvement to develop pak choi cultivars with altered flowering characteristics suited to different cultivation requirements. For example, late-flowering cultivars may be preferred in high-temperature regions or for leafy vegetable production to maximize output and quality. On the other hand, plants that produce flowers early may be better for breeding or producing seeds. Genome-editing methods such as CRISPR/Cas can be used to alter the natural alleles of *BcFPA* in different germplasm collections. This can help create cultivars with optimized flowering traits. Future studies should further evaluate natural allelic variation of *BcFPA* and its potential application in breeding programs.

## Conclusions

In summary, this study identified the autonomous pathway regulator *BcFPA* as a candidate gene associated with natural flowering-time variation in pak choi. FPA proteins exhibit conserved flowering-promoting effects across species while harboring species-specific sequence mutations that drive functional divergence. Within Brassicaceae, *BcFPA* retains the typical domain structure of FPA family genes but carries a unique functional polymorphism controlling bolting phenotype. It functions via the conserved *BcFLC-BcFT* regulatory module, integrating autonomous and photoperiod flowering signals. The locus and linked molecular markers identified here provide practical resources for molecular breeding aimed at controlling premature bolting in commercial pak choi production. Future research will focus on exploring natural allelic diversity of *BcFPA* in wider pak choi germplasm and dissecting its upstream and downstream interacting regulatory factors to further clarify the complete flowering regulatory network of leafy Brassica vegetables.

## Supplementary Information

Supplementary Material 1. Table S1: Segregation analysis of flowering time phenotypes in plant materials. Table S2: Basic statistics of BSA-seq data. Table S3: Statistical table with information about candidate interval. Table S4: Annotated genes located in the 2.62-Mb candidate interval on chromosome A09. Table S5: InDel markers within the 4.26-kb candidate interval. Table S6: Primers used in the study

Supplementary Material 2. Figure S1: The *Brap0046500.1* DNA sequence in M2-001. Figure S2: The *Brap0046500.1* DNA sequence in M1-006. Figure S3: Sequence alignment of the *BcFPA* CDS sequences from M1-006 and M2-001.

## Data Availability

The datasets generated and/or analyzed during the current study are available in the NCBI Sequence Read Archive (SRA) repository under accession number PRJNA1475153. Additional data supporting the findings of this study are included within the article and its supplementary information files.
