# Supplementary material for: Identification and functional validation of the BcFPA, a candidate regulator of flowering time in pak choi
Source: BMC Plant Biol. 2026 Jul 28;26:1824. doi: 10.1186/s12870-026-09561-z (PMC13693014; doi:10.1186/s12870-026-09561-z)
Supplement: Supplementary file 1 [file 12870_2026_9561_MOESM2_ESM.docx]

ATGGCACCAGTAATGAAGCCTTCAAGAGGCCGTGAACCCGACGGATCTGGATTCCAATCCACCAATCTCTGGGTCGGTAGTCTGACCTCCGATACTACCGAATCCGATCTCGCCGATCTGTTCGGAAGGTTCGGCGATATCGACAGGCTCACCGCGTATTCTTCCCGCTGCTTCGCCTTTATCTACTACAGGCGCGTTGAGGAAGCCGTAGCTGCAAAGGAAGCTCTTCAAGGCGCCAATCTGAACGGGAGTCGCATTAAAATCGAATTCGCACGACCGGttcgttctcatctctacagtctcttgttagtggttagatcgggaatttcacagatcatgagtgccacgtatgcttgatttgtggatccacaaatgattgcttcttcttatggataacagttttgctataatgcttaatctcataaagattgattatgaacttcaaatgcgttcaagtttctgaaacgtaattagtatcttaatccttcggattagacttggtgaatcccagagttttcagattctgcaaaatgtttctcgtgtttgagatgtaaatttggaagtgatctttgtagttcctctagtcatcagagatttgtaacagtaacgctaccaacatggtgggaggttttttttggttatcgtagaccatgaaagactttgccagcatcttatgctttctgctcatcttagtgggtccaagggttggtttatttagtaatgtgccaaatttaaaatatgatcaacacagaggacatagttctcttctatttatggaaatgattctcctcaagtcatgttgtgtgtttatgggaataattaaatgggaatttattactacaatctatgaattagtaaccgattttgttttgtagtgatttatgtgtgtttaaaaaggttctcttcatgacaaccaggaaagccaattctggatgttgttaagatgtattttattggcttcttatggaaattatttgtgtgcaggCAAAACCTTGTAAGAGTCTTTGGGTGGGTGGAATCAGCTCGAGTGTCCCGAAGGATGTACTCGAGGCAGAGTTCAGGAAGTTTGGGGAAATCGAAAGTTTCAGGTTTCTTAAGGATCGCAAGACGGCTTTTATTGACTATTTTGACGTGGACGATGCTGTACAGGCAAAGACCATGAACGGGAAGCGACTAGGTGGTAGCTTTTTGCGAGTTGATTTTCTTCGGTCACAAGGGCCAAGAAAAGtaagcagcctcttgtcgtttttttttttttgcttttacattagaataagcttcagtaatcattttgttagaagcttctactgaatgcgttaggtttcatgatttgtataagactgccatgactagaagctttctatatctacctgtgtcacgtgtttccatttttatttcttctgcgtttatctttttcatatctatgttgtagaatctgtttaatgactctgaaattacgtgacattcttatctctgtaggAACCACGGCTTGGATCCGAAGATGGCAGGGATGGCAGTTTGAGTGCTAAGCACCAGgttagcctttatcggctttcatgtttatagtttagaaagctttcggcttgccaggttcctgagttcaaatttttatatgcttaacaaCAGTATACTCACTCATCTGGAGATGGCCAAGCAAGTAACGTTCTGTGGATTGGGTACCCTCCTTCTGTTCAGATAGACGAACGACTGCTGCACAATTCCATGATTCTTTTTGGTGAGATTGAGAGATGTGTTAGCTATCCATCAAGGCATTTCTCACTTGTGGAATTTAGAAGCATTGAGGAAGCTCGCTGTGCTAAGGAAGGACTACAGGGAAGGTTATTCAACGATCCAAGAATCACAATCATGTACCAAAACGATAACATTCCCCCTGGACGGGGAGATGATACCGGTTTTTATTCTGGTGTCAAACGGTCAAGACCTGAGATGTTCATCAGTGATCCTCCGTACATATCGTCACCACATTCCAGTGTGGTTCTTGACCCTGTGAGGCCCTTCCGAGGTAGCATGGAACGTTTTGGTCCAGAATATGGCGATGTGGTTGGCAACGAAGGAAGCTGGAAAAGGCCATCTCCTAGTGGAGCTGGAATACTCCCGTCTCCCGCACCAAGTAGAAGGCTACCCATTCGGTCAAACCGTGGTTCTTGGGAAGGATATGATCCTGCTCAGTTGGATAGAGAACCTAAAAGAACTCGTAGAGATGGATCTGTAGATGCTTTTCCTCCGATGGGTGTAGATGATCGTATCACTGGGTTGGATAGGACATATCGACGTAGACCAGGCCGTGGTTTCCCTGATACTGATTTCATTTGGAGAGGAGTCATAGCCAAAGGTGGAACTCCTGTTTGTCATGCTCGTTGTGTACCTATTGGAAAGGGGATTGAATCTGAACTgtgagtaacattttcatgcactttagcctttttttttgccttcacttatgtaggataagttttagttcatagattagaagttatcttatctgttgattcttttagatgttgtgatctgaaaatggattttgcatagtgtttgattcgattcattgataacaagtaccgaatggattactgattatcggtttttggaattggttaatagCCCTGAGATGGTCAATTGTTCAGCACGAACGGGTTTGGATATGCTTGCCAAACACTATACGGAAGCAATTGGATTTGAAATCGTTTTCTTCTTACCAGACAGCGAAGACGACTTCGCGTCTTATACTGAATTTCTCCAATATCTTGGTTCAAAGAATAGAGCAGGTGTTGCCAAATTAGATGATGGAACCACTTTATTCTTGGTTCCACCATCAGATTTCTTAACTGATGTGCTCAAAGTATCCGGTCGAGAGAGGCTATACGGTGTTGTTCTCAAATTGCCACCACCACCGGTTCCTGCAGCTTTATCGTATAGACAAGAATCTCATACCGTTCCTCCACACTATATGGATCAGTCTCGAGATTCGCCTGCCAGTGCCAATCACGGTTTGTACGCTGCTAGGGAAGATCAAGCCTGGGGTATACAGGAACCAATGAAGCCTGTTGCTGTACATAACACTGCAACGCCTCAAGCCGGGGTTAGTCTAACGCCTGAGCTAATAGCCACTCTTGCCTCCTTGCTCCCAACAAATTCTCAAGCAGAAAGTCACCAAGCCGTTGGATTATCCAATGGAGAAGCTCCATCACAAGCCTGGAATAGAGATCAATCAACGGTTTCTGACCCATCAAATCTATCGTTTCAACAATTAGGCAATCAGTACAATCCGGTGGGGCAACTGCCTCCTCCTCCGGTGCACTATCCTCCAGTGTCAAGCACACCGAGCTACTCCGGTGGAATGGTACAAGGTGGTATGCAATACCAGGCGGCGTTACCTCCACCACCATCGGATAATTACTCCATGTACTCTCAAGGTTCACATCAAGCTCAGTATCAGCATGAAACTCCCATGCTTAACCAAAACTACGTATCGGTCCCTGGAGCTACGAACCCTGGTTTGCTTTGTTATCAGGGTTATCAGCAAGGTAACTATCACGGTCTGACAAATAATCAAGCTTATAACGAAAATCCTTCCCATTCTCAAATTGCGATGCCACCACCAACACAACAGCAGCCACAGCTTGCGGCTGGTCAAGGTACATCAGATGGTGAGGACGACAAGAGCCAGCAATATCAGTCGACTCTACAGCTTGCAGCTAACCTTCTTCAACAGATTCAGCAACGGCAGCAACTGCCATCGAATGCTTCGACTGGACAAGGGCCTTGA

**Figure S1. The *Brap0046500.1* DNA sequence in M2-001**

Uppercase letters indicate exons, lowercase letters denote introns, yellow-highlighted regions correspond to the InDel fragment, and red marks represent the positions of amplification primers.

ATGGCACCAGTAATGAAGCCTTCAAGAGGCCGTGAACCAGACGGATCTGGATTCCAATCCACCAATCTCTGGGTCGGCAGTCTGACCCCCGATACTACCGAATCCGATCTCGCCGATCTGTTCGGAAGGTTCGGCGATATCGACAGGCTCACCGCCTATTCTTCCCGTTGCTTCGCGTTTATCTACTTCAGGCGCGTTGAGGAAGCCGTTGCCGCTAAGGAAGCTCTTCAAGGCGCCAATCTGAACGGGAGTCGCATTAAAATCGAATTCGCACGACCGGttcgttctgatcatcgcgtttcttcctttaactctgatacagtctcttgttaatggttagatcgggaatttcacagatcatgagtgccacgtatgcttgataaatgattgctattgctattgctctgtagatcataatttgaaatatgatcaacgcaggattttttttttgttgactttgaagaggacatagttctctggcttctatttatggaaatgattctcctcaagtcatgttgtgtgtttatggtagtaattaaatgtgaatttttattacaatctttgaattagtaactgattttgttttgtagttttatttatgtgtttaaaaaggttctcttcatgacaaccaggaaagccaattctggatgttgttaagatgtattttattggcttcttatggaaattatttatgtgcaggCAAAACCTTGTAAGAGTCTTTGGGTTGGTGGAATCAGCTCGAGTGTCCCGAAGGATGTACTCGAGGCAGAGTTCAGGAAGTTTGGGGAAATCGAAAGTTTCAGGTTTCTTAAGGATCGCAAGACGTCTTTTATTGACTATTTTGACGTGGACGATGCTGTACAGGCAAAGACCATGAACGGGAAGCGACTAGGTGGTAGCTTTTTGCGAGTTGATTTTCTTCGGTCACAAGGGCCAAGAAAAGtaagcatcctctttgtctttttttactagaataagttttggtttccatcttgttagaagcttctaccaagtgcgttaggtttcatgatttgcataggattgcatggctagaagctttctatatctacctgtgtcacgtgactccatttttatttcttatgcgtttacttttttgatatctatgttatagaatctgctaaatgactctgaaattacgtgacattctcatctctgtaggAACCACGGCTTGGCTCCGAAGATGGCAGGGATGGCAGTTTGAGTGCTAAGCACCAGgttagccttttactggtgatatgtgcaatcctttatcggctttcatgtttatagtttagaaagctctcggcttgccaagttcctgatagttcaaatttttatatgtttaacagCAGTATACTCACTCACCTAGAGATGGCCAAGCAAGCAACGTTCTGTGGATTGGGTACCCTCCTTCTGTTCAAATAGACGAACGACTGCTGCACAATTCCATGATTCTTTTTGGTGAGATTGAGAGATGTGTTAGCTATCCATCAAGGCATTTCTCACTTGTGGAATTTAGAAGCATTGAGGAAGCTCGCTGTGCTAAGGAAGGATTACAGGGAAGGTTATTCAACGATCCAAGAATCACAATCATGTACCAAAACGATAATAGTCCTCCTGGAAGGGGAGATGATACCGGTGTTTATCCTGGTGTCAAACGACCAAGACCTGATATGTTCATCAATGATCCTCCGTACATATCGTCACCACATTCCAGTGTGGTTCTTGACCCTGTGAGGCCCTTCCGAGGTAGCATGGAACGTTTTGGTCCAGAATATGGCGATGTGGTTGGCAACGAAGGAAGCTGGAGAAGGCCATCTCCTAGTGGAGCTGGAATACTCCCGTCTCCCGCACCAAGTAGAAGGCTACCCATTCGGTCAAACCGTGGTTCTTGGGAAGGATATGATCCTGCTCAGTTGGATAGAGAACCTAAAAGAACTCGTAGAGATGGATCTGTAGATGCTTTTCCTCCGAGGGGTGTAGATGATCGTATCACTGGGTTGGATAGGACATATCGACGTAGACCAGGCCGTGGTTTCCCTGATACTGATTTCATTTGGAGAGGAGTCATAGCCAAAGGTGGAACTCCTGTTTGTCATGCTCGTTGTGTACCTATTGGAAAGGGGATTGAATCTGAACTgtgagtaacattttcatgcactttagccttttctgccttcacttatgtaggataagttttagttcatagattagaagttgtcttatccgttgattcttttagatgttgcatagtgtttgattctattcattgataacaagtaccgaatggattactgattatcggtttttggaatggttaatagCCCTGAGATGGTCAATTGTTCAGCACGAACGGGTTTGGATATGCTTGCCAAACACTATACCGAAGCAATTGGATTTGAAATCGTTTTCTTCTTACCAGACAGCGAAGACGACTTCGCGTCTTATACTGAATTTCTCCAATATCTTGGTTCAAAGAATAGAGCAGGTGTTGCCAAATTAGATGATGGAACCACTTTGTTCTTGGTTCCACCATCAGATTTCTTAACTGATGTCCTCAAAGTATCCGGTCGAGAGAGGCTATACGGTGTTGTTCTCAAATTGCCTCCACCACCTGTTCCTGCAGCTTTATCGTATAGACAAGAATCTCAGACCATTCCTCCACCCTATTTGGATCAATCTCGAGATTCGCCTGCCGGTGCCAGTCACGGTTTGTACGCTGCTAGGGAAGATCAAGCCTGGGGTATACAGGAACCAATGAAGCCTGTTGCTGTACATAACACTGCAACGCCTCAATCCGGGGTTAGTCTAACGCCTGAGCTAATAGCCACTCTTGCCTCCTTGCTCCCAACAAATTCTCAACCAGAAAGTCACCAAGCCGTTGGAGAAGCTCCATCACAAGCCTGGAATAGAGATCAATCAATGGTTTCTGACCCATCAAATCACTCGTTTCAACAATTAGGCAATCAGTACAATCCGGTGGGGCAACTGCCTCCTCCCCCTCCTCCGGTGCATTATCCTCCAGTGTCAAGCACACCGAGCTACTCTGGTGGAATGGTACAAGGTGGTATGCAATACCAGGCGGCGTTACCTCCACTACCATCGGATAATTACTCCATGTACTCTCAAGGTTCACATCAAGCTCAGTTTCAGCATGAAACTCCCATGCTTAACCAAAACTACGTATCTGTCCCTGGAGCTGCGAACCCTGGTTTGCTTGCTTATCAGGGTTATCAGCAAGGTAACTATCACGGTCTGACAAATAATCAAGCCTATAACGAAAATCCTTCCCATTCTCAAATTGCGATGCCACCACCAACACAGCAGCAGCCACAGCTTGCGGCTGGTCAAGGTACATCAGATGGTGAGGTCGACAAGAGCCAGCAATATCAGTCTACTCTACAGCTTGCGGCTAACCTTCTTCAACAGATTCAGCAACGGCAGCAACTGCCATCAAATGATTCGACTGGACAAGGGCCTTGA

**Figure S2. The *Brap0046500.1* DNA sequence in M1-006**

Uppercase letters indicate exons, lowercase letters denote introns, yellow-highlighted regions correspond to the InDel fragment, and red marks represent the positions of amplification primers.

**Figure S3. Sequence alignment of the *BcFPA* CDS sequences from M1-006 and M2-001**
